# Primary cell and tissue cultures of human choroid plexus epithelial cells

**DOI:** 10.3389/fncel.2026.1827493

**Published:** 2026-08-31

**Authors:** Ronja Bihlmaier, Melanie Scharr, Peter H. Neckel, Bernhard Hirt, Andreas F. Mack

**Affiliations:** Institute of Clinical Anatomy and Cell Analysis, Eberhard Karls Universität Tübingen, Tübingen, Germany

**Keywords:** aquaporin-4, cerebrospinal fluid, choroid plexus, ependyma, human tissue culture, glial cells

## Abstract

Choroid plexus epithelial cells (CPEC) are implicated in cerebrospinal fluid (CSF) production and the site of the inner blood-cerebrospinal fluid barrier. CPEC are specialized ependymal cells with characteristic cell junctions, intermediate filaments, and transport protein expression, including aquaporins. We have recently found that, in addition to aquaporin-1, a subset of CPEC in the human brain express aquaporin-4 (AQP4), the main water channel in the brain that has been implicated in water homeostasis and glymphatic system function. We therefore aimed to establish primary cultures from human CP to study AQP4 expression. We collected CP tissue from human body donors *post mortem* to grow CPEC *in vitro*. We successfully established two primary culture models for human CP tissue. In an organ culture model, small pieces of CP tissue were explanted on PTFE membranes. In a second model, cells from CP were dissociated and seeded on either laminin or collagen substrate. We monitored cell and tissue growth and investigated protein expression by immunofluorescence. Tissue integrity of explant cultures was maintained throughout the cultivation period of several weeks. Immunofluorescence signals for zonula occludens protein-1 (ZO1), AQP1, and Na/K-ATPase were present although the expression patterns became increasingly irregular and non-polar over time. In dissociation cultures, cells attached and grew very slowly initially but reached confluency after several weeks. Cultures were passaged up to 6 times and could be cryopreserved. Application of cytosine arabinoside suppressed the growth of macrophages and most likely fibroblasts. These primary dissociation cultures were identified as epithelial by their morphology and positive immunofluorescence for transthyretin, vimentin. Na/K-ATPase, ZO-1, cytokeratin, and AQP1. In both culture models, cells expressed AQP4. In dissociation cultures, AQP4 appeared to colocalize with ZO-1 at cell–cell contact sites in many CPEC but showed separate localizations at high resolution. These studies show that primary organ and cell cultures can be generated from human *post mortem* CP tissue suitable to investigate regulation and localization of AQP4. Together, these culture models provide valuable tools to investigate the proteins involved in CSF production and glymphatic clearance, relevant for the pathogenesis of neurodegenerative diseases.

## Introduction

The choroid plexus (CP) is a specialized structure found in all brain ventricles (I-IV), and commonly accepted as the location where the bulk of the cerebrospinal fluid (CSF) is produced. In the human brain, the CP of the lateral ventricles extends over several centimeters on the ventricular surface (except for the anterior and occipital horn) with the largest thickness at the posterior curvature. The CP surface is formed by epithelial cells (choroid plexus epithelial cells; CPEC) with a basal lamina, and an underlying stroma containing blood vessels and varying amounts of stromal cells and extracellular matrix in between. CPEC express a number of ion channels and transport proteins believed to be involved in the production of CSF (Praetorius and Damkier, 2017). In addition, they form tight junctions which are the basis for the blood-cerebrospinal fluid barrier, reviewed in (Rasmussen et al., 2022; Abbott et al., 2006; Engelhardt and Ransohoff, 2012).

The single cell layer formed by CPEC is continuous with the ependymal lining of the brain ventricles. However, CPEC differ from ventricular ependymal cells in their barrier function, expression of transport proteins, and apical differentiation (Wolburg and Paulus, 2010; Peters, 1974; Spector et al., 2015). For example, murine CPEC express the water channel aquaporin-1 (AQP1) apically, whereas the adjacent ependymal cells express aquaporin-4 (AQP4) which is also found in astrocytes (Mack and Wolburg, 2013). In the human CP however, we recently found AQP4 also in a substantial number of CPEC, possibly due to age of the body donors the tissue was derived from (Deffner et al., 2022). Thus, the human CP tissue differs from murine models not only in size and shape but also in protein expression. This in turn is likely to have an impact on CSF production.

The observation that not all human CPEC were positive for AQP4 raises the question how this heterogenous expression of aquaporins is regulated. As a first step to make human CP tissue experimentally accessible, we aimed to maintain human primary CPEC *in vitro.* Generally, human adult CP tissue is difficult to obtain due to its central location in the brain and rare indications for surgical excision (Redzic, 2013), and there have only been few attempts of culturing human CP as primary cultures (e.g.Wroblewska et al., 1981). Human CPEC have been cultured as cell lines, derived from CP papilloma (Speidel et al., 2022; Ishiwata et al., 2005; Bernd et al., 2015). Additionally, a stem cell derived human CP organoid model has recently been developed (Pellegrini et al., 2020). However, immortalized CP cell line cultures are not well suited for certain aspects of CP biology (Lazarevic and Engelhardt, 2016). In this study, we explored the option to attain cell cultures from human *post mortem* CP tissue generously provided by body donors, and asked the question whether these cultures would maintain the expression of AQP1 and AQP4. We used two different approaches for human primary CP culture studies: explant cultures on membrane inserts, and dissociation cultures. We report here that human CPEC from *post mortem* tissue can be maintained over several weeks to months in cell culture and continue to express characteristic proteins. These models will be valuable to study many aspects of CP biology including its role in neurodegenerative diseases.

## Materials and methods

### Human CP primary cell culture protocol

Human CP tissue was obtained from individuals who generously donated their body to the Institute of Clinical Anatomy and Cell Analysis in Tübingen (Table 1). Approval for this project was obtained from the Ethics Committee of the Medical Faculty of the University of Tübingen under the project number 532/2025BO2. Additionally, for comparison of the stainings we used fixed human CP tissue previously obtained (see Bihlmaier et al., 2023; Supplementary Table 1). CP tissue for cell culture was collected within 12 h *post mortem*. After removal of the brain, the CPs from the lateral and fourth ventricles were carefully separated from the adjacent brain tissue with a scalpel and transferred into a 50 mL tube filled with sterile preparation buffer (Hanks’ balanced salt solution (HBSS) without Ca^2+^/Mg^2+^ (Sigma-Aldrich, Taufkirchen, Germany), penicillin (100 U/mL; PAA, Cambridge, UK), streptomycin (100 mg/mL; Sigma-Aldrich, Taufkirchen, Germany)).

**Table 1:** Human specimen.

ID	Age	Sex	*PMI* (h)	Cause of death*
H101	80	w	10	Cardiac arrest
H102	92	m	8	Acute abdomen
H103	79	m	12	Pneumonia & heart failure

* cause of death as stated in the official death certificate.

### Explant culture

For the tissue cultures, small pieces of CP tissue (2–3 mm in length) were excised from epithelial regions and placed on polytetrafluoroethylene (PTFE) membrane inserts ((Millicell® Cell Culture Inserts), Merck Millipore, Billerica, MA, USA) in cell culture medium consisting of a mixture of Dulbecco’s modified Eagle’s medium and Ham’s F12 medium (DMEM) (Life technologies, Darmstadt, Germany), at a ratio of 1:1 containing 10% (v/v) fetal calf serum ((FCS), Biochrom, Berlin, Germany), penicillin (100 U/mL), streptomycin (100 mg/mL) and L-glutamine (2 mM; Sigma-Aldrich, Taufkirchen, Germany). To maintain optimal conditions, 1.5 mL of culture medium was added to each well, ensuring that the medium level exceeded the height of the insert membrane to prevent the membrane from drying out. An overview of CP tissue used from each body donor and replicates of cultures, see Table 2.

**Table 2:** Overview of replicates for culture experiments for each tissue donor.

H101 (collected tissue: 2x lateral ventricle CP, third ventricle CP, fourth ventricle CP)
Dissociation cultures
4x ½ (one per choroid plexus) 12-well plate coated with collagen
Passaged 6 times onto collagen coated plates in different sizes, glass bottom dishes and ibidi slides
Longest in vitro time: 146 days
4x ½ (one per choroid plexus) 12-well plate coated with laminin
Passaged 6 times onto laminin coated plates of different sizes, glass bottom dishes and ibidi slides
Longest in vitro time: 146 days
Explant cultures
3 × 6-Well plate with prepared tissue explants (5 per Well) on PTFA inserts
Longest in vitro time: 40 days
H102 (collected tissue: 2x lateral ventricle CP, fourth ventricle CP)
Dissociation cultures
1× 12-well plate coated with collagen
Passaged 6 times onto collagen coated plates in different sizes, glass bottom dishes and ibidi slides
Longest in vitro time: 91 days
1× 12-well plate coated with laminin
Passaged 4 times onto laminin coated plates in different sizes, glass bottom dishes and ibidi slides
Longest in vitro time: 83 days
Explant cultures
3 × 6-Well plate with preparated tissue explants (5 per Well). on PTFA inserts
Longest in vitro time: 34 days
H103 (collected tissue: 2x lateral ventricle CP, fourth ventricle CP)
Dissociation cultures
1× 12-well plate coated with collagen
Passaged 3 times onto collagen coated plates in different sizes, glass bottom dishes and ibidi slides
Longest in vitro time: 44 days
1× 12-well plate coated with laminin
Passaged 3 times onto laminin coated plates in different sizes, glass bottom dishes and ibidi slides
Longest in vitro time: 44 days
Additionally: cryopreservation at −80 °C and passaged for the 4th time, additional in vitro time: 4 days
Explant cultures
6 × 6-Well plate with preparated tissue explants (5 per Well) on PTFA inserts
Longest in vitro time: 7 days

### Dissociation culture

For dissociation cultures, CP tissue was further dissected, separating epithelial regions from the stroma and vasculature. The epithelial parts were then transferred into a 50 mL tube with HBSS solution containing Ca2 + and Mg2+. For dissociation, an enzyme mixture of collagenase type XI (750 μg/), dispase type II (250 μg/mL) and DNase (0.05% (w/v); all from Sigma Aldrich Chemie GmbH, Taufkirchen, Germany) was added and incubated at 37 °C for 20 min. During this incubation period, trituration was performed every 5 min and an additional 0.05% DNase solution was added. After 20 min of enzymatic digestion, the process was stopped by the addition of 10% (v/v) FCS. The cells were then centrifuged at 200 g for 6 min in the tube. The supernatant was carefully removed and the cell pellet was resuspended in HBSS without Ca2+ and Mg2+. This washing step was repeated and, afterwards, cells were resuspended in culture medium. Before seeding, the suspension was additionally passed through a cell strainer (FalconTM Cell Strainer ∅100 μm Becton, Dickinson and Company, Franklin Lakes, NJ, USA). Due to the extensive presence of cell fragments and aggregations in human *post mortem* samples, cell counting was not possible before the first seeding. Therefore, all cells were initially plated. Cell counts were performed on subsequent passages using a Neubauer counting chamber and trypan blue stain exclusion. Cells were then seeded in a concentration of 10,000 cells/cm^2^. The cells were either seeded on collagen (collagen type 1 [(rat tail), 3,3 ug/ml; BD Biosciences, Heidelberg, Germany)] or laminin (Sigma Aldrich Chemie GmbH, Taufkirchen, Germany) coated culture plates (Costar® 12-Well Dish, Corning Inc., Corning, NY, USA) using the same cell culture medium as for the explant cultures and maintained at 37 °C with 5% CO_2_. Additionally, cytosine arabinoside (AraC, 20 μM; Sigma Aldrich Chemie GmbH, Taufkirchen, Germany) was added for the first 3 days, to prevent growth of non-epithelial cultures such as macrophages and fibroblasts. The culture medium was replaced based on the metabolic rates of the cells, typically every 3 to 5 days.

After reaching confluency cells were passaged to new dishes. To detach the cells from the culture plates, preheated trypsin (37 °C; gibco®, Life Technologies, now: Thermo Fisher Scientific, Waltham, MA, USA) was applied for 6 to 8 min. The cells were resuspended in culture medium, and seeded again at a concentration of approximately 10,000 cells per cm^2^ on either collagen- or laminin- coated culture plates. For high power microscopy optics, some cells were cultivated on glass bottom dishes (Ibidi® *μ*-Slide 8 Well (high glass bottom), ibidi GmbH, Gräfelfing, Germany). See Table 2 for an overview and replicates performed for dissociation cultures.

For cryopreservation, the detached CP epithelial cells were resuspended in 1 mL of freezing medium (Dulbecco’s Modified Eagle Medium with Ham’s F12 medium (DMEM) 1:1; Life technologies, Darmstadt, Germany) containing 20% (v/v) FCS, Biochrom, Berlin, Germany) and 10% Dimethyl sulphoxide ((DMSO), AppliChem GmbH (Darmstadt, Germany). Approximately one million cells were cryopreserved in 1 mL of freezing medium. The tubes were then placed in a freezing container and stored overnight at −80 °C. On the following day, the tubes were transferred to a nitrogen tank for long-term storage.

### Immunohistochemistry

Immunostains were carried out on freshly fixed choroid plexus tissue, explant cultures, and dissociation cultures. All tissue samples were fixed in 4% paraformaldehyde (PFA, AppliChem, Darmstadt, Germany). Tissue samples not intended for culturing were fixed overnight, cultures were fixed in PFA for 20 min after removal of medium and brief rinses in phosphate buffered salt solution (PBS). The immediately fixed control samples were processed for cryostat sections as previously described (Deffner et al., 2022). In short, tissue was cryoprotected in 30% sucrose frozen in TissueTek (Sakura, Staufen, Germany) and sectioned at a cryostat at 18 μm. Immunostains on cryosections and culture samples followed our standard lab protocols of incubation times and washing steps (Deffner et al., 2022). Briefly, after 3×10 minutes washes in PBS, sections were incubated for 90 min in a blocking solution containing either goat serum (Biochrom, Berlin, Germany) or donkey serum (Biochrom, Berlin, Germany), depending on the secondary antibodies for the specific stainings. Primary antibodies detecting AQP1, AQP4, Na/K-ATPase, Transthyretin, and Vimentin as well as phalloidin diluted in PBST (PBS with 0.03% TritonX, see Table 3 for dilutions and source) were applied at 4 °C overnight. After three washes in PBS, secondary antibodies (Table 4) were applied for 90 min at room temperature together with either the nuclear stain DRAQ5 (1:1000; Thermo Fisher Scientific, Waltham, MA, USA) or DAPI (1:2000; Carl Roth GmbH & Co. KG, Karlsruhe, Germany). After additional washes with PBS samples were coverslipped and mounted with Mowiol (Carl Roth GmbH & Co. KG, Karlsruhe, Germany). For tissue culture stainings, incubation times were extended depending on the size of the tissue. Each staining combination was performed at least three times with cultures from different tissue donors.

**Table 3:** Primary antibodies.

Antibody	Supplier	Cat No.	Host animal	Dilution
ZO-1	Thermo Fisher Scientific (Waltham, MA, USA)	33–9,100	Mouse	1:50
AQP-4	Santa Cruz Biotechnology (Dallas, TX, USA)	sc-20812	Rabbit	1:100
AQP-4	Santa Cruz Biotechnology (Dallas, TX, USA)	sc-9888	Goat	1:100
AQP-1	Thermo Fisher Scientific (Waltham, MA, USA)	PA5-78805	Rabbit	1:100
AQP-1	Santa Cruz Biotechnology (Dallas, TX, USA)	sc-32737	Mouse	1:100
TTR	Sigma Aldrich Chemie GmbH (Taufkirchen, Germany)	SAB3500378	Chicken	1:250
CD-31	Abcam (Cambridge, England)	abcam28364	Rabbit	1:100
GFAP	Santa Cruz Biotechnology (Dallas, TX, USA)	sc-58755	Mouse	1:100
NKA	Hybridoma Bank (Iowa, USA)	a6F	Mouse	1:100
Iba 1	FUJIFILM Wako Chemicals Europe GmbH (Neuss, Germany)	019–19,741	Rabbit	1:100
NKCC1	Abcam (Cambridge, UK)	ab59791	Rabbit	1:100
HuCD	Invitrogen (CA, USA) now: Thermo Fisher Scientific (Waltham, MA, USA)	A21271	Mouse	1:100
Vimentin	Santa Cruz Biotechnology (Dallas, TX, USA)	ec-6260	Mouse	1:100

Cytokeratin Invitrogen (CA, USA) MA1-10325 Mouse 1:30 now: Thermo Fisher Scientific (Waltham, MA, USA).

**Table 4:** Secondary antibodies.

Antibody	Supplier	Host animal	Dilution
Anti-rabbit Alexa 546	Invitrogen (CA, USA)	Goat	1:400
Anti-rabbit Alexa 488	Invitrogen (CA, USA)	Goat	1:400
Anti-rabbit Alexa 660	Invitrogen (CA, USA)	Goat	1:400
Anti-mouse Alexa 488	Invitrogen (CA, USA)	Goat	1:400
Anti-mouse Alexa 546	Invitrogen (CA, USA)	Goat	1:400
Anti-mouse Alexa 660	Invitrogen (CA, USA)	Goat	1:400
Anti-Mouse Alexa 546	Invitrogen (CA, USA)	Donkey	1:400
Anti-Goat Alexa 488	Invitrogen (CA, USA)	Donkey	1:400
Anti-Rabbit Alexa 647	Invitrogen (CA, USA)	Donkey	1:400
Anti-Rabbit Alexa 546	Invitrogen (CA, USA)	Donkey	1:400
Anti-chicken Alexa 488	Invitrogen (CA, USA)	Goat	1:400

### Imaging

Images of live cultures were taken on an Axio Imager Z1 equipped with an Apotome module (Carl Zeiss Jena/Oberkochen, Germany), using phase contrast optics. For fluorescence detection, either a confocal microscope system (LSM510 Meta, LSM Exciter, or LSM 900 Airyscan, Zeiss, Germany) with laser lines at 488, 543 and 633 nm for excitation and appropriate filter sets, or the Apotome Axio Imager were used. All images were initially recorded with the Zeiss system software and image plates were assembled with Photoshop CS2 (Adobe Inc., Mountain View, CA, USA).

## Results

In this study we aimed to establish primary cultures for human CPEC in order to investigate functional mechanisms in CP tissue. In particular, we were interested in the possible expression of functional proteins involved in CSF homeostasis like aquaporins in these cultures for further studies on their regulation. We used two main *in vitro* approaches: We cultured (1) small pieces of CP tissue as explants, and (2) dissociated cells. In addition, several culture parameters such as substrate and media supplements were tested.

### Explant cultures

**Figure 1:**
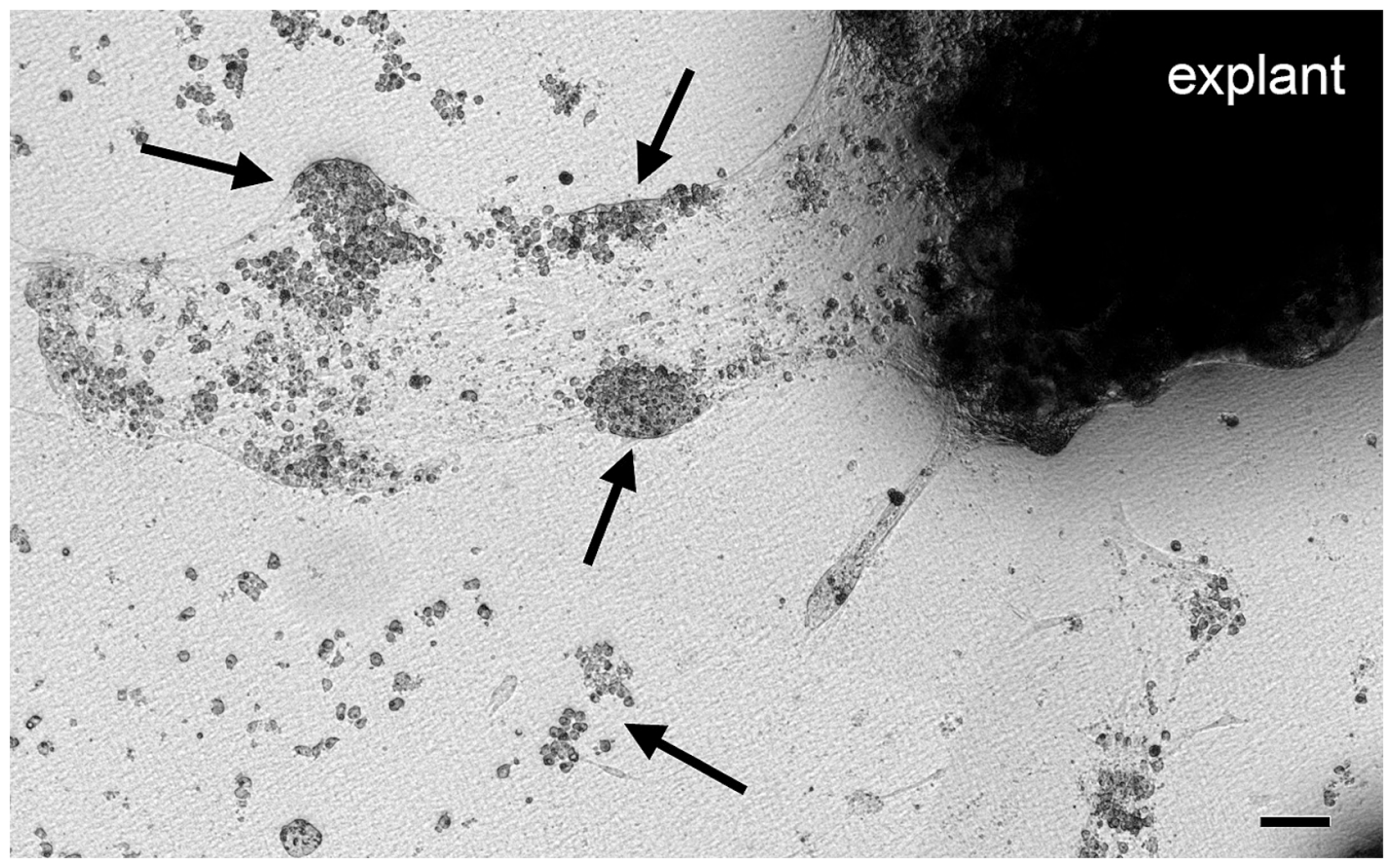
Transmitted light image of human CP explant culture of body donor H101 on a PTFE membrane insert after 19 days *in vitro*. Groups of cells (arrows) were found at the border of the explanted tissue. This indicates cell growth and migration. Scale bar = 100 μm.

Human CP tissues from three different body donors (IDs: H101, H102, H103) were cultured as tissue explants. Initially, we tried to maintain explants on laminin and collagen coated dishes (as used for dissociation cultures). However, whole tissue pieces did not attach well although few single cells did. We therefore used PTFE membrane inserts to keep explants *in vitro*. The explants were closely monitored over time up to 40 days in culture, and we observed expanding cell groups at the edges of the tissue. This indicated not only viability of the explanted *post mortem* tissue but also cell growth and proliferation (Figure 1; supplementary Figure 1).

**Figure 2:**
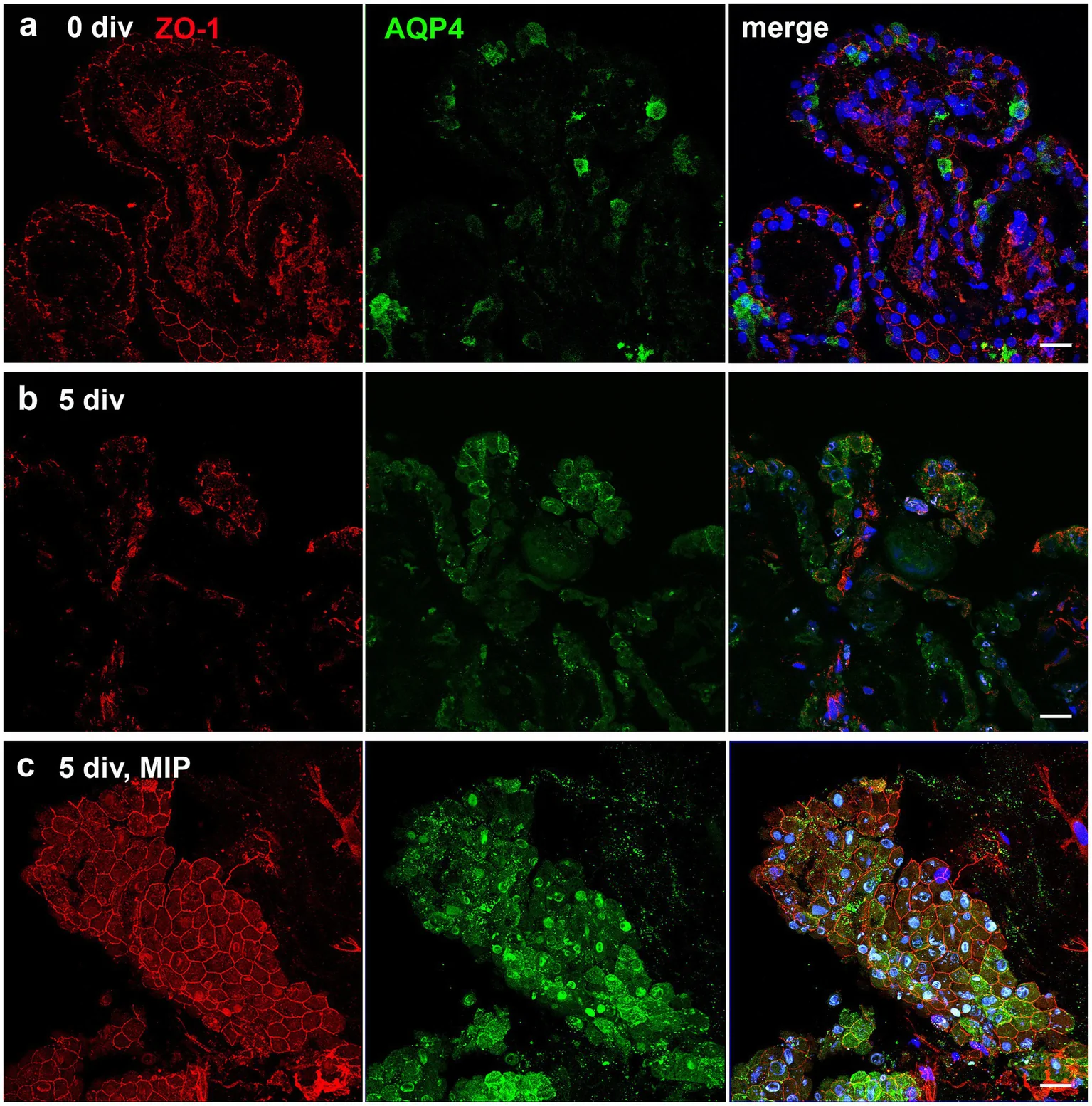
ZO-1 and AQP4 expression in CP tissue and explants. **(a)** Confocal image of cryostat section of fixed human CP showing discontinuous ZO-1 staining (red) at epithelial cell borders and several AQP4-positive (green) CPEC. Nuclei are stained with DRAQ5 (blue). **(b)** Confocal image of cryostat section of human CP culture explants after 5 days *in vitro* (div), showing discontinuous ZO-1 expression, and several cells with AQP4-positive signals, located mostly at the basolateral membrane. Nuclei are stained with DRAQ5 (blue). **(c)** In some explant regions, continuous ZO-1 expression after 5 div is present depicted in a maximum intensity projection of a confocal image stack. In addition, the region shows a higher number of AQP4-positive cells with varying expression patterns, membrane location in some cells and cytoplasmic expression in others. Nuclei are stained with DAPI (blue). Cultured tissue was taken from body donor H101 in panel **(b)**, and from body donor H102 in panel **(c)**. Scale bars = 20 μm.

**Figure 3:**
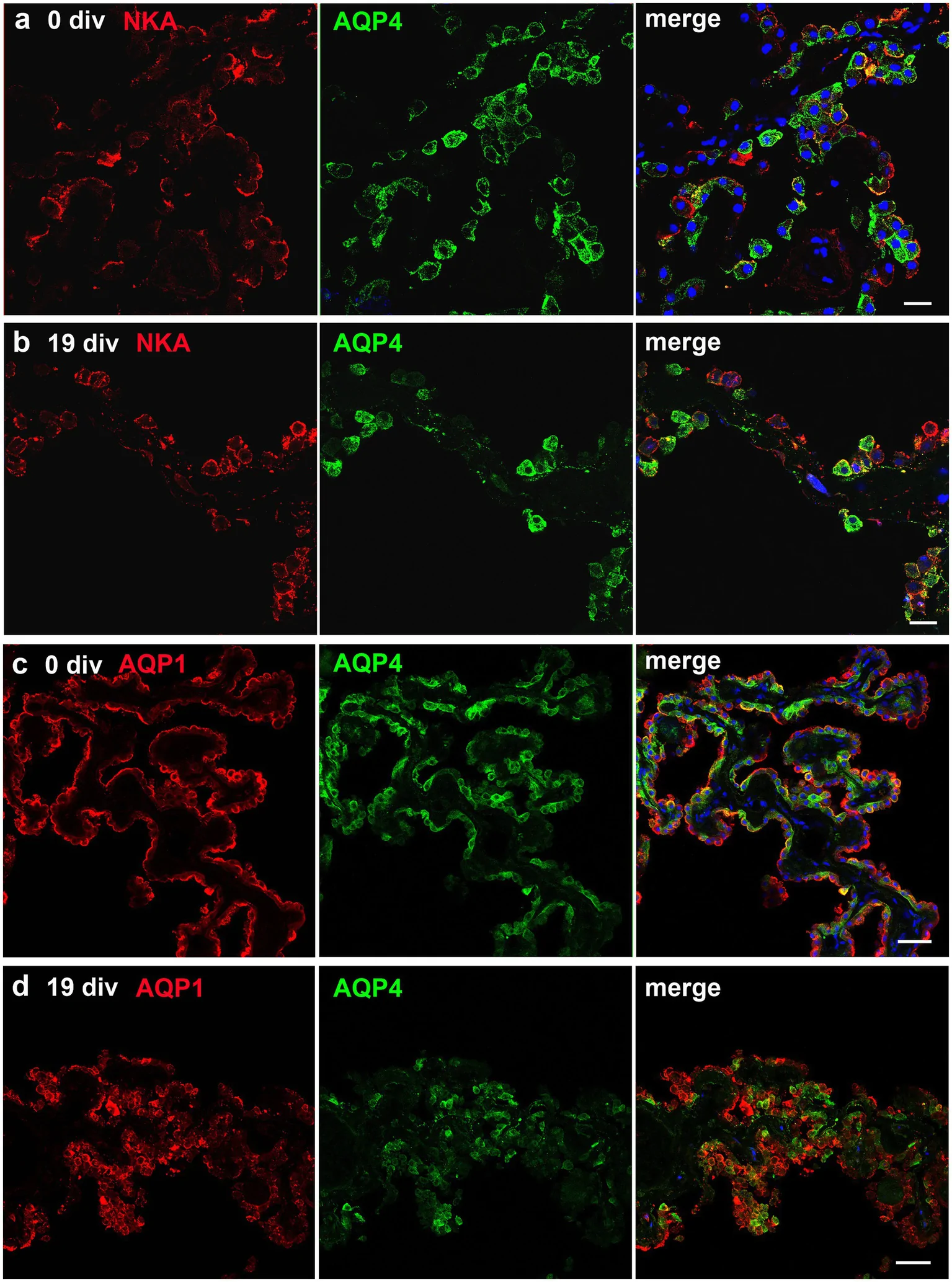
AQP1 and Na/K-ATPase (NKA) expression in combination with AQP4 in CP tissue and explants. Panel **(a,c)** are immunostains of immediately fixed tissue (0 div), **(b,d)** explant cultures (19 div). **(a)** Cell-typical apical NKA (red) expression can be observed on most CPEC. AQP4 (green) expression varies between the CPEC. **(b)** The expression of both proteins is maintained throughout the culture period. In some CPEC apical NKA expression is maintained, whereas in others the polarity of the expression is lost, similar to AQP4. Nuclei are stained with DRAQ5 (blue). **(c)** Apical AQP1 (red) expression can be observed on most CPEC. AQP4 (green) expression pattern varies between the CPEC. **(d)** The expression of both proteins is maintained throughout the culture period although fewer cells display the typical apical AQP1 expression. Nuclei are stained with DRAQ5 (blue). Cultured tissue was taken from body donor H101. Scale bars **(a,b)**: 20 μm; **(c,d)**: 50 μm.

To find out whether the cultured tissue maintains its integrity we performed immunohistochemistry for zonula occludens protein-1 (ZO-1), Na/K ATPase (NKA) and AQP1 (Figures 2, 3). Since we had recently found AQP4 in human CPEC, we combined the stains with immunolabeling for this water channel. Samples were fixed after different periods of cultivation. Figure 2 shows ZO-1 staining of CP tissue cultured for 5 days compared to immediately fixed CP tissue (0 div). In both, fixed and cultured human CP tissues, the ZO-1 expression pattern showed heterogeneity across regions, with fragmented ZO-1 staining in some areas and continuous ZO-1 staining in others. This ZO-1 immunostaining pattern confirmed the epithelial organization of the cells on the filter membranes. However, the epithelial integrity of the cultured tissue varied substantially between regions and donors. Yet, cells in tissue cultures maintained the cell-typical expression of AQP1 and NKA at least for 19 div, although the expression patterns became increasingly irregular and non-polar over time (Figures 3b,d). This indicates that CP tissue can be kept in culture but the heterogeneity also shows the limits for quantitative assessment of this approach.

Despite quantitative variations, all human body donor samples showed AQP4-positive CPEC in the fixed control tissue confirming previous results (Deffner et al., 2022), (Figures 2a, 3a,c). Remarkably, cultured CP tissue maintained this AQP4 expression (Figures 2b,c, 3b,d) at least up to 19 div. Similar to AQP4 expression in fixed CP, the number of AQP4-positive cells and the intensity of the fluorescence signal in the cultured tissue varied substantially between regions and donors. In some CPEC, AQP4 was exclusively expressed in the basolateral membrane (Figure 2b), whereas in others it remained membrane-associated but non-polar (Figure 3b). Additionally, several CPEC showed a cytoplasmatic AQP4 expression pattern (Figure 2c). Co-staining of AQP4 and ZO-1 was performed to investigate the possible influence of ZO-1 integrity on the AQP4 expression pattern but no apparently visible correlation was observed. Likewise, we did not observe any staining pattern that would indicate a relationship between AQP4 and NKA, resp. AQP1.

### Dissociation cultures

Dissociation cultures of human primary CPEC were prepared and established from CP tissues of the same three body donors (H101, H102, H103). Compared to murine cultures (data not shown), dissociated cells from the human CP tissue needed much longer to adhere to the substrate (at least 4 days, see below), yet grew sufficiently to be passaged several times.

Initially, CP tissue was obtained from the lateral, third, and fourth ventricles and cultured separately. Dissociation cultures from the lateral ventricle CP reached confluency substantially faster than those from the third and fourth ventricle CP (supplementary Figure 5). For establishing the cultures, we therefore focused on tissue from the lateral ventricle CP. Two methods were employed to obtain dissociation: (1) enzymatic digestion, and (2) the acquisition of cells from floating CP tissue. The latter approach implied that CPEC detached from the tissue and adhered to the culture substrate. While both methods yielded monolayer cultures, the floating culture gave inconsistent results in terms of cell numbers and survival. We therefore analyzed enzymatically digested human CPEC cultures in subsequent experiments. The cells were cultured on two types of substrate coating: laminin and collagen.

**Figure 4:**
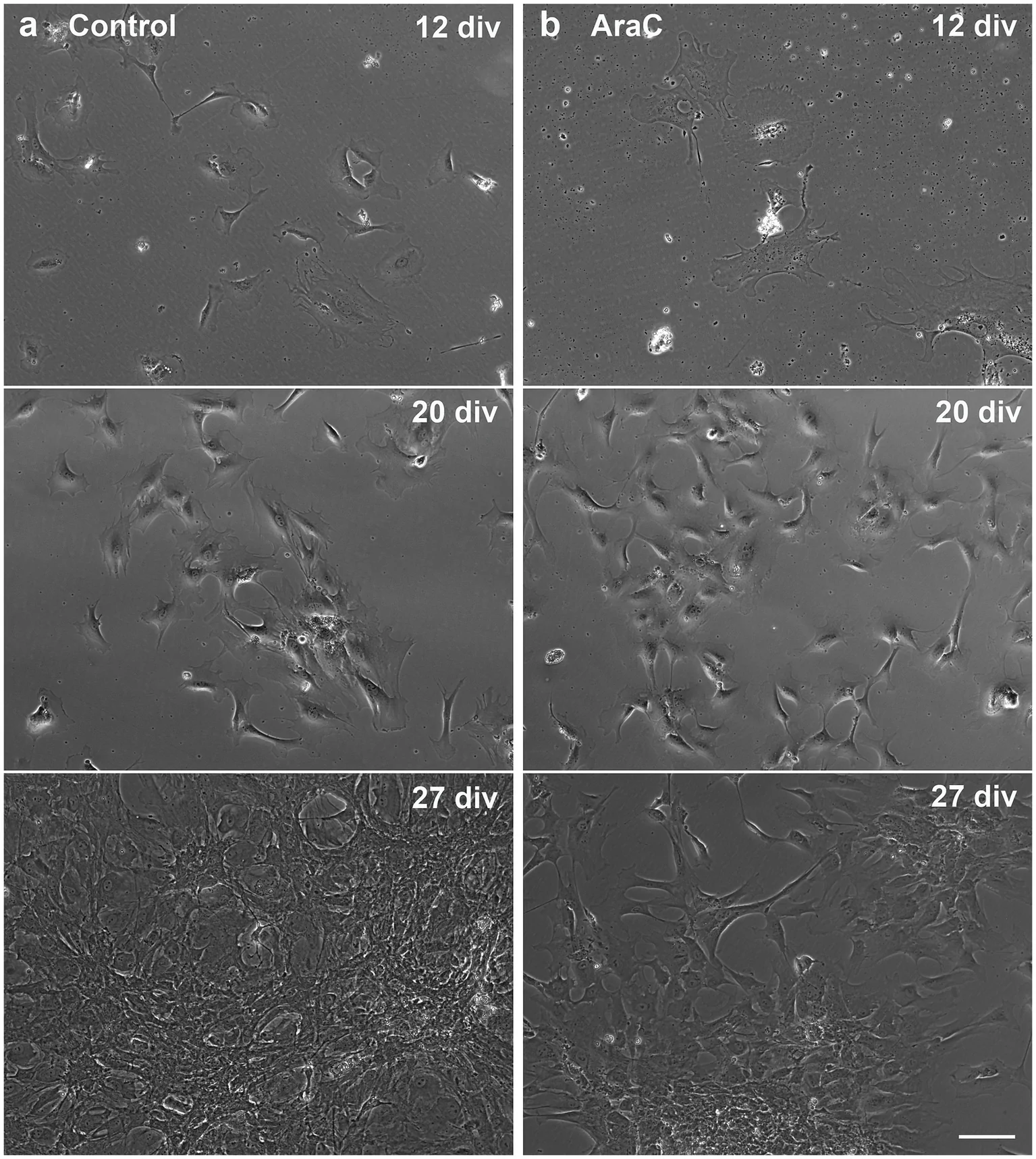
Growth of primary dissociation cultures - effect of AraC. Phase contrast images of cultures on laminin substrate of body donor H101 at 12, 20, and 27 div. **(a)** Untreated control group, **(b)** cultures treated with AraC for the first three days after seeding. In the early stages, both groups show similar growth but untreated cells are more heterogeneous in cell morphology, including small spindle-shaped cells. After several weeks, cells in the control group exhibit higher cell densities, with cells overgrowing each other. Scale bar: 100 μm.

**Figure 5:**
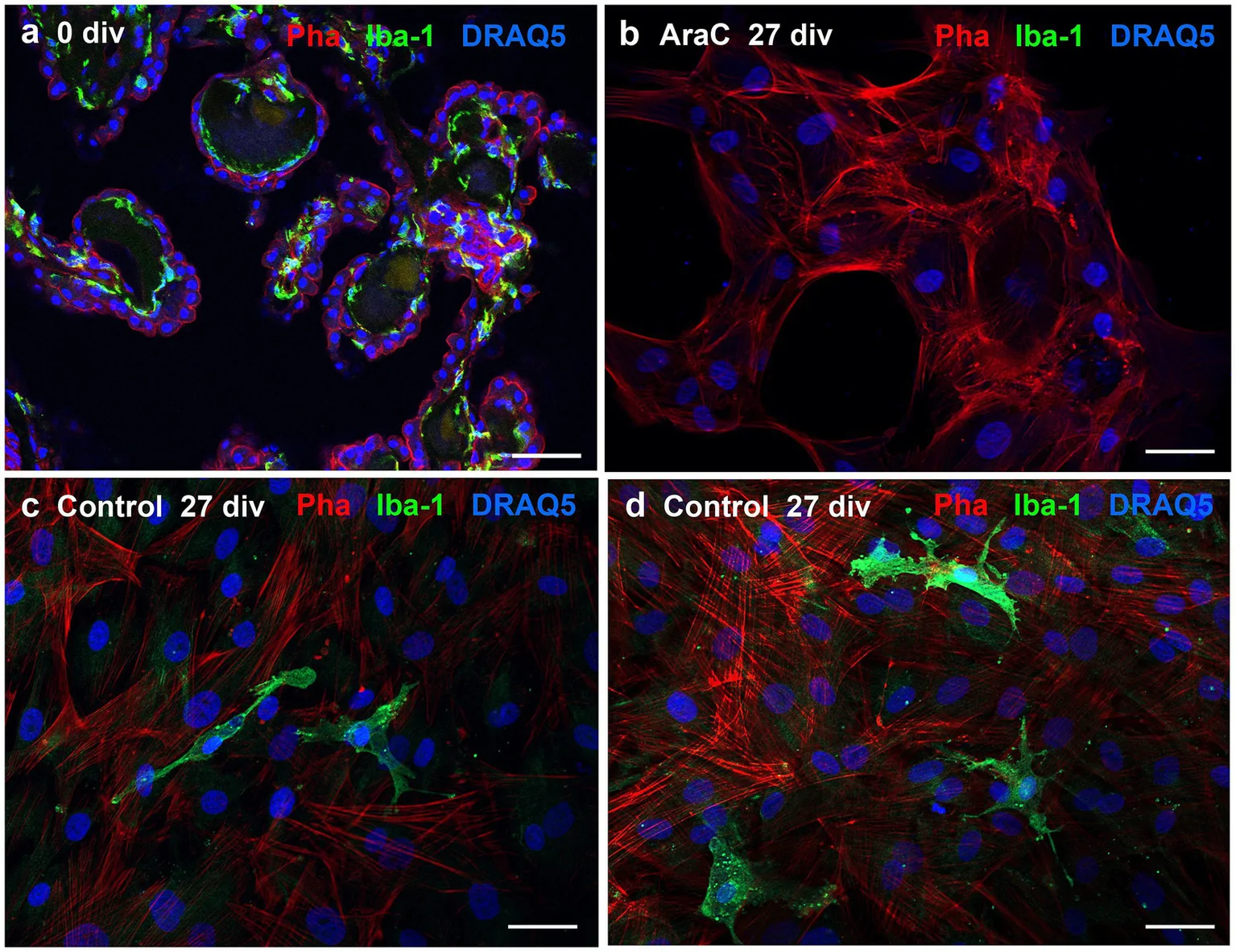
Effect of AraC on Iba-1 positive cells. Iba-1 and phalloidin (actin) staining in freshly fixed, sectioned CP tissue shows the abundance of macrophages in the CP stroma **(a)**. Dissociation cultures treated with AraC for the first three days, followed by three weeks *in vitro* **(b)**. Examples of different untreated dissociation cultures (no AraC) as controls **(c,d)**. Iba-1 staining (green) reveals the presence of several macrophages in untreated cultures whereas no Iba-1 positive cells are found after AraC treatment indicating that it effectively eliminates non-epithelial cells. Pha: phalloidin stain (red) labels actin fibers. Nuclei are stained with DRAQ5 (blue). Cultured tissue was taken from body donor H101. Scale bars: 50 μm.

Previous studies of mammalian CP tissue (Schroten et al., 2012) suggested that cytosine arabinoside (AraC) prevented fibroblast growth in culture. We tested and confirmed this result by applying AraC to the medium for 3 days. Only minimal differences were observed initially, yet after several weeks in culture, the control group (no AraC) showed a higher cell density and less homogeneous morphology (Figure 4). These observations indicated a selective inhibitory effect of AraC on the proliferation of specific cell types (especially on macrophages, see below). To identify possible non-epithelial cells in the control culture, we immunolabeled with astroglial (GFAP), endothelial (CD-31) and macrophage markers (Iba-1). GFAP and CD-31 staining showed no positive cells in any of the cultures (Supplementary Figure 4). Interestingly, in cultures without AraC, several Iba-1-positive macrophages could clearly be identified. However, no Iba-1-positive cells were observed in AraC treated cultures indicating an inhibitory effect on macrophages (Figure 5).

Dissociated cells reached confluency 3–4 weeks after first plating. They could then be passaged several times. Attempts to improve cell growth by the addition of growth factors (IGF-1) as suggested for murine postnatal cultures (Barkho and Monuki, 2015) were not successful. This needs to be investigated in further studies.

**Figure 6:**
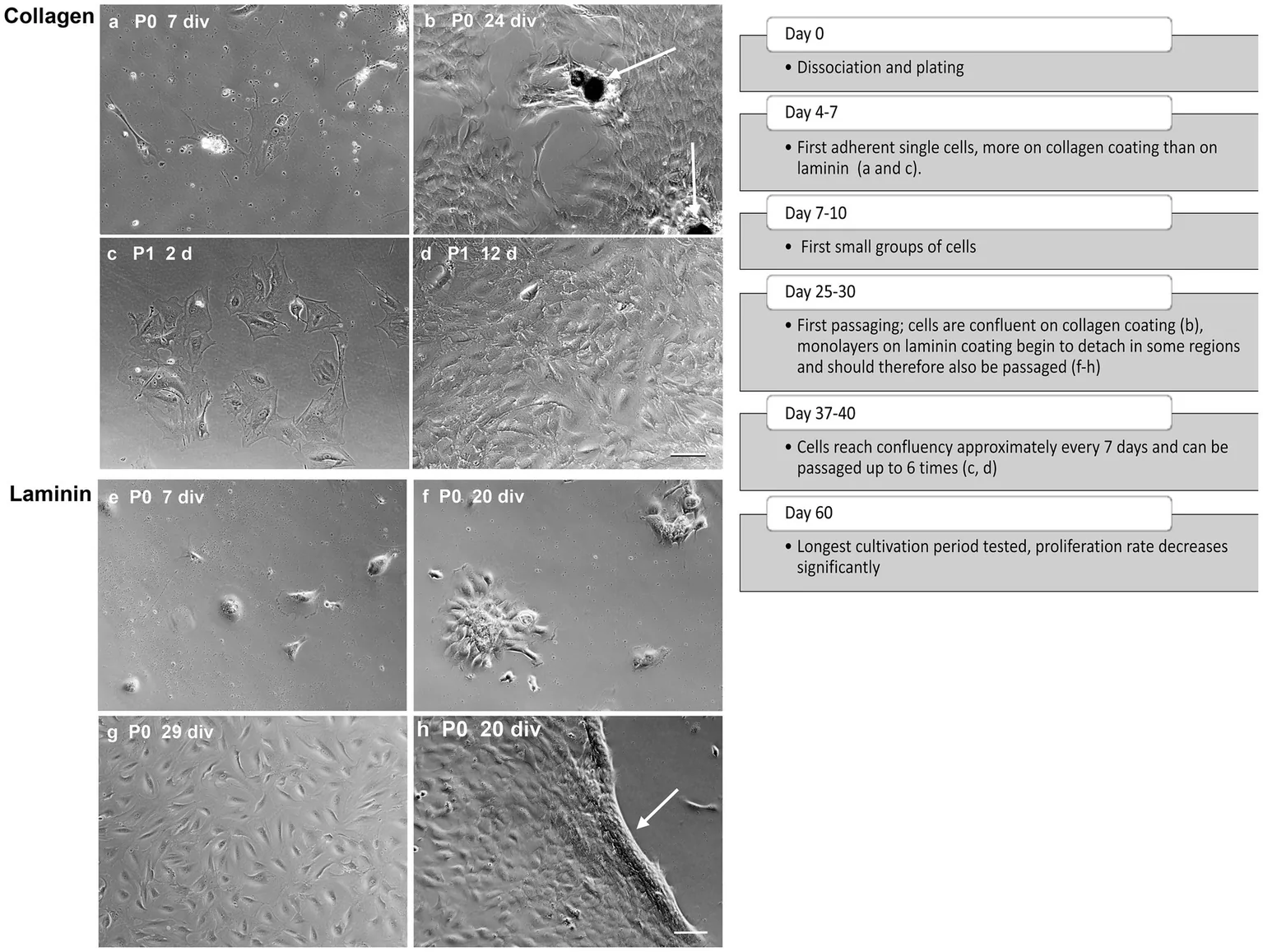
Growth of dissociation cultures on collagen vs. laminin. **(a–d)** CPEC on collagen coating. **(a)** After 7 days *in vitro* (div) showing heterogeneous morphologies and several processes. **(b)** CPEC grow in groups and become more homogeneous. While cells continue to grow as a monolayer in some regions, aggregates of cells are observed in others (arrows). **(c,d)** After the first passage, the cultures are viable and show groups of cuboidal cells forming a more homogeneous monolayer over time than after the initial plating. Scale bar: 100 μm. **(e–h)** CPEC on laminin coating. **(e)** After 7 div on laminin coating, showing a more homogeneous morphology with fewer processes than on collagen coating. **(f)** Cells grow in dense groups, leaving cell-free areas in the dish, especially before the first passage. **(g)** The cultures build a dense monolayer and reach confluency. **(h)** Occasionally, the dense monolayer detaches from the laminin surface and retracts (arrow). The images are typical examples from all three body donors, specifically: **(f–h)** from body donor H101, **(a,c,g)** from body donor H102, and **(b,d)** from body donor H103. Scale bar: 100 μm. On the right, a short timeline of steps and observations in primary CP dissociation cultures is shown.

Figure 6 shows examples of dissociated cells on collagen and laminin substrate and provides a short timeline of the steps occurring in the established human primary cultures. After tissue dissociation, it took approximately 4 to 7 days for the first cells to adhere to the culture surface. On collagen substrate, cells adhered better initially, resulting in more detectable cells compared to cultures on laminin-coated dishes. At later stages, cells and growth on collagen and laminin were similar.

Before the first passage, the cultures contained cell debris and non-viable cells which were difficult to remove but did not inhibit cell proliferation. The newly dissociated cells showed a heterogenous morphology, often with numerous processes. This morphological diversity was more pronounced in cells cultured on collagen-coated plates than on laminin-coated plates (Figures 6a, c). By day 7 to 10, cells began to aggregate into small groups on both collagen and laminin coatings, and cells had shorter or no processes. Cells on collagen-coated plates were more evenly distributed over the plates, whereas cells on laminin-coated plates grew in dense clusters (Figure 6f) to finally form a monolayer with typical epithelial morphology. After longer cultivation (25–30 days), this dense layer detached from the laminin-coated plates in some regions and formed an aggregated cell mass (Figure 6h). The empty plate region was slowly repopulated. Typically, from approximately 20 mL of excised CP tissue volume, we could spread the cells over 2 to 4 12-well plates in P0 cultures. For passaging, cells were seeded at a density of approximately 10,000 cells per cm^2^ on an expanded culture surface area of approximately 150 cm^2^ in various dishes. Cultures reached confluency already after about one week after re-plating (Figure 6). Once established, the cultures remained stable for at least five passages, however, thereafter cell growth became slower and ceased. Furthermore, we examined whether the passaged cells could be cryopreserved for subsequent use. After freezing, storage at −80 °C for several days, and thawing, the cells were seeded again and readily grew again to confluency (Supplementary Figure 6). Thus, cell culture could effectively be restored after cryopreservation.

**Figure 7:**
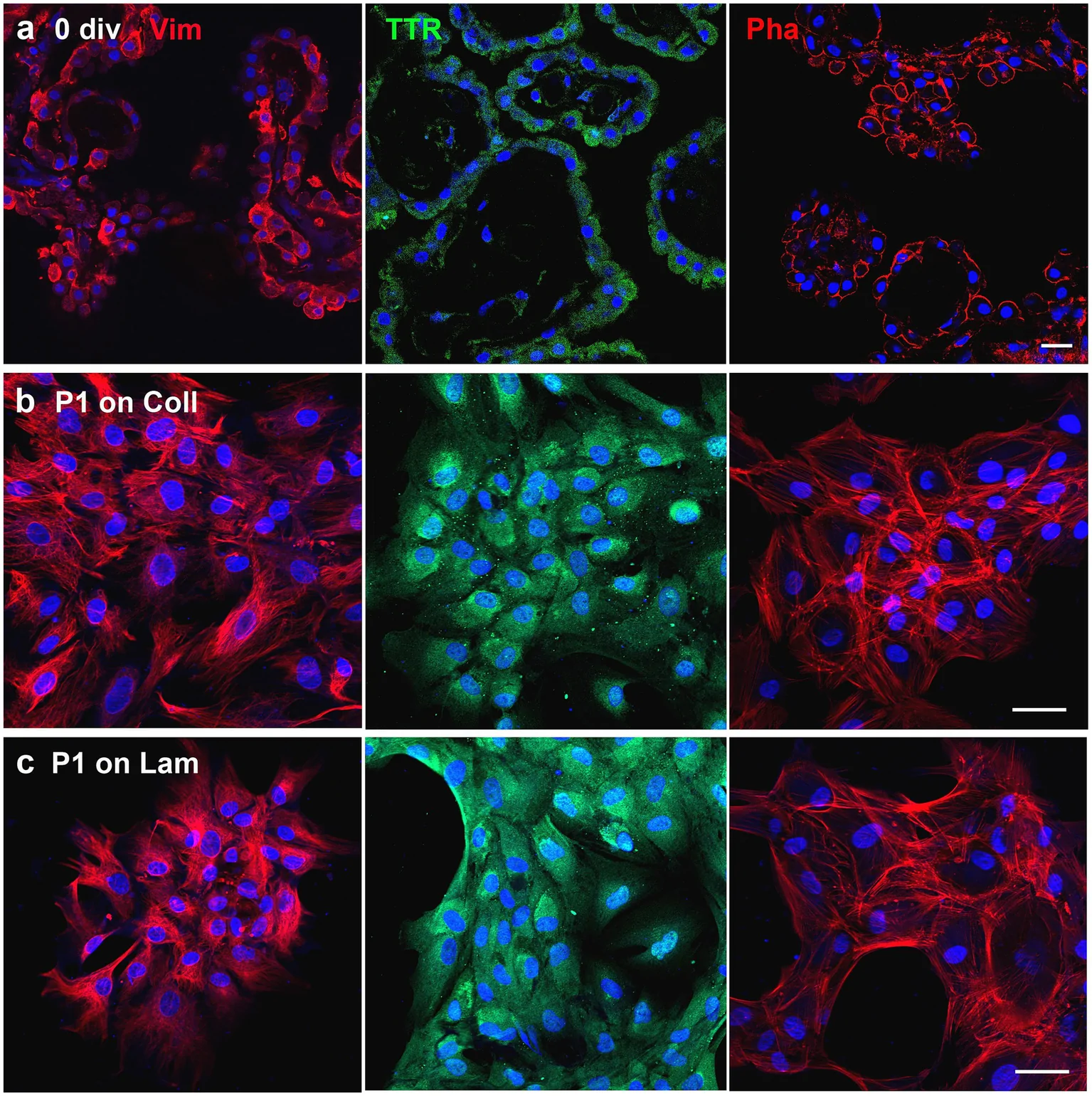
Characteristic protein expression in human primary dissociation cultures. CPEC stained for Vimentin (Vim), Transthyretin (TTR), and Actin (Phalloidin, Pha) as indicated. **(a)** Cryostat section of immediately fixed CP tissue (0 div). **(b)** Primary dissociation culture on collagen, and **(c)** on laminin after the first passage (P1). The stains for Vim, TTR, and Pha display epithelial cell-typical expression patterns and are similar in collagen and laminin-coated dishes. Nuclei are stained with DRAQ5 (blue). Stained cultures were prepared from body donor H102. Scale bars: **(a)** 20 μm, **(b,c)** 50 μm.

**Figure 8:**
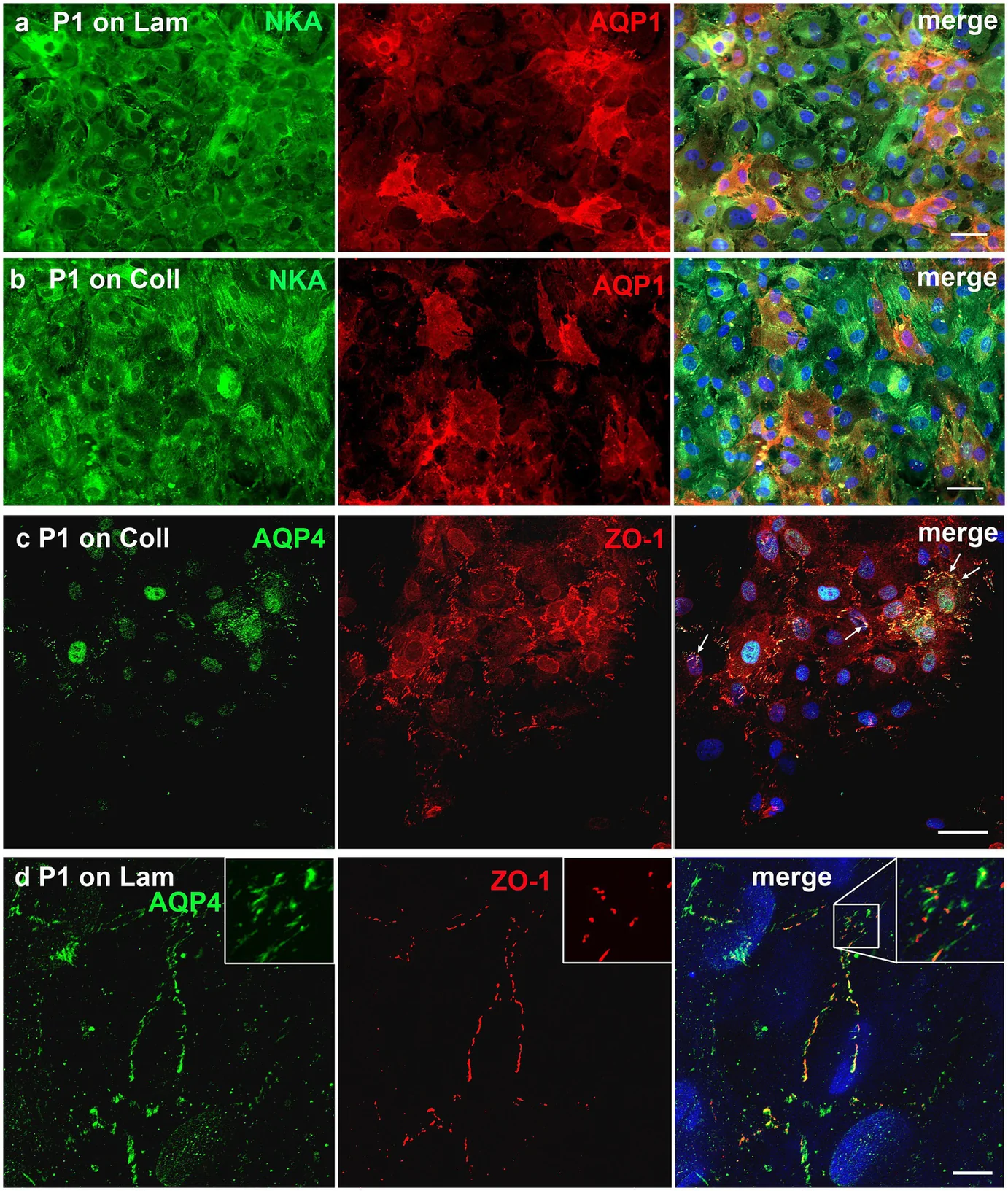
Expression of Na/K-ATPase (NKA), AQP1, AQP4, and ZO1 in dissociation cultures. Cultured CPEC on laminin, **(a,d)**, and on collagen, **(b,c)**, after the first passage (P1). **(a,b)** The stains for NKA and AQP1 show that typical functional proteins are expressed, yet alterations in the expression patterns are observed, indicating a loss of polarization. AQP1 immunofluorescence intensity varies strongly between cells, whereas the NKA expression pattern is more homogeneous. **(c,d)** Co-staining for AQP4 and ZO-1. At low resolution, AQP4 appears to co-localize with ZO-1 at cell–cell contact sites in many CPEC **(c)**. However, the two expression patterns show separate localizations at high resolution (**d**, Airy scan, magnified inserts). This indicates a distinct regulation of the localization to the membrane for ZO-1 and AQP4 compared to AQP1 and NKA in these cultures. Nuclei are stained with DRAQ5 (blue). Cultures were prepared from body donor H102. Scale bars: 50 μm in **(a–c)**, 10 μm in **(d)**.

To verify the epithelial origin of the cells and asses their potential functionality *in vitro*, we stained for cytoskeletal and characteristic markers of CPEC (vimentin, actin, transthyretin, ZO-1, cytokeratin), transport and channel proteins (NKA, NKCC1, AQP1, AQP4), and markers for other cell types possibly occurring (GFAP for astrocytes, Iba1 for macrophages, CD-31 for endothelial cells, and HuCD for neurons). Figure 7 shows stainings of cultures after the first passage with actin-binding phalloidin, and antibodies against vimentin and transthyretin (TTR), in comparison to fixed tissue (0 div). Cells were readily positive for these markers in both, cultures on collagen and laminin. Therefore, the substrate did not seem to affect expression patterns. For TTR, more cells were unequivocally stained in cultures than in fixed tissue. Cultured cells were also positive for NKA and AQP1 although the expression did not seem to be restricted to the cell membrane (Figures 8a,b). Interestingly, the intensity of the immunofluorescence signal for AQP1 expression varied significantly between cells, whereas the NKA staining showed consistent intensities. In addition, we stained for the epithelial marker pan-cytokeratin that labeled most cells in dissociated cell cultures (Supplementary Figure 2).

Importantly in the context of our previous studies, AQP4 expressed in some cells of the human CP *in vivo,* was also detected in dissociated CPEC cultured on both collagen and laminin coatings (Figures 8c, d). AQP4 expression was generally heterogenous, with the number of positive cells varying between different regions of the same plate, across passages, and between cells from different body donors (Supplementary Figure 3; Supplementary Table 1). At low resolution, AQP4 appeared to colocalize with ZO-1 at cell–cell contact sites in several CPEC (arrows in Figure 8c). However, the two stainings showed distinct expression localizations at high resolution (insert in Figure 8d). The substrate did not seem to influence AQP4 expression as we had hypothesized since the staining patterns were similar in cells on collagen and laminin coating (Figures 8c,d).

Immunostainings for GFAP, Iba-1, CD-31, and HuCD to test for other cell types did not reveal any positive cells in the dissociation cultures (Supplementary Figure 4).

## Discussion

In this report we show that cells derived from *post mortem* human CP can be maintained and grown *in vitro.* We established two different approaches to keep CPEC in culture. We found that adult *post mortem* CPEC are viable in explant cultures and can be expanded in dissociation cultures. This is remarkable because primary cell cultures from the CNS including CP are commonly generated from juvenile or embryonic tissue samples (Redzic, 2013; Delery and MacLean, 2019). Additionally, we were able to show that these CPEC maintain their protein expression *in vitro*, including AQP4 recently located in the CP. This provides new possibilities to study its expression but the heterogeneity also shows the limits for quantitative assessment of this approach.

### Advantages and challenges of primary cell cultures

Different culture approaches (primary cultures, cell lines, 3D-cultures, and organoids) have been used to study CP cells. Cell lines offer advantages such as providing a pure and consistent population with reproducible results, easy handling, cost-effectiveness and, especially concerning CP material, accessibility. However, many immortalized CP cell line cultures have shown aberrations from the characteristics of primary CPEC *in vitro*, exhibiting changes in morphology and properties, such as a lack of adherens junction proteins resulting in a poor barrier formation compared to primary cultures of rat or porcine CP (Szmydynger-Chodobska et al., 2007; Strazielle and Ghersi-Egea, 2011). Moreover, for investigations of immune cell migration, primary murine CP culture proved to be more suitable than a tumour-derived cell line lacking claudins and cadherins (Lazarevic and Engelhardt, 2016). Alterations in protein expression were reported in immortalized CPEC (HCPEpiC, commercially available from ScienCell Res. Lab.) compared to primary cells (Lallai et al., 2020). Despite these challenges, certain cell lines, such as the HiBCPP cell line derived from human CP papilloma, have demonstrated expression of relevant marker genes, and dense and continuous tight junctions under certain conditions, making them suitable for barrier studies (Ishiwata et al., 2005; Speidel et al., 2022). Primary cultures of CPEC have been established from a variety of animal species including rodents (Villalobos et al., 1997; Strazielle and Ghersi-Egea, 1999) bovine (Crook et al., 1981), porcine (Haselbach et al., 2001) and non-human primate species (Delery and MacLean, 2019), yet due to the differences between human and animal CP, these approaches have limited value to study the biology of the human CP. Primary human CPEC (offered by ScienCell Res. Lab.) are from fetal origin with very limited availability and raises ethical concerns in many countries. Interestingly, Pellegrini et al. managed to develop a human CP organoid model from pluripotent stem cells (Pellegrini et al., 2020) which will be useful for future studies. Our approach provides a more straightforward and faster way to investigate human CPEC. This is especially valuable for studies on age- or disease-related changes.

It will be interesting to investigate CP samples from patients with neurodegenerative diseases, since, for example, several changes in CP tissue have been associated with Aβ accumulation and Alzheimer’s disease. In particular, age-related changes observed in the CP such as epithelial cell atrophy, thickening of the basal lamina, increased deposits (as Biondi ring tangles) and stromal fibrosis are significantly more pronounced in Alzheimer’s disease (Serot et al., 2003; Serot et al., 2001; Wen et al., 1999).

### Cell growth

Although there is little information regarding proliferation in the adult human CP, our results show that it is possible to stimulate human CPEC to expand in primary cultures. This implies cell division and is clear evidence that CPEC have the capacity to proliferate. CPEC are specialized ependymal cells and as such are classified as macroglial cells which are derived from neuroepithelial cells together with neurons (Rowitch and Kriegstein, 2010). However, CPEC are unique to the CNS in exhibiting typical epithelial properties including the formation of a basal lamina and cell polarity. Although one characteristic of epithelial tissue in general is its regenerative capacity, proliferation in the CP epithelium is reported to occur only at a very low rate if at all, mostly described for developmental stages (Liddelow, 2015). Proliferative and differentiation capacity was implied in studies of cultured CPEC from young mice grafted into the spinal cord where some cells expressed astrocytic markers (Kitada et al., 2001). Our results suggest that the slow turnover rate of CPEC can be enhanced by proliferative culture conditions. Despite the longer time required for human CPEC cultures to expand and reach confluency after the initial seeding, they grew sufficiently to undergo multiple passages in medium supplemented with 10% FCS. Although suggested by previous studies in murine CP cultures (Barkho and Monuki, 2015), the addition of IGF-1 could not substitute for FCS nor did it show any additional proliferation effect in combination with FCS in the adult *post mortem* cells. Notably and important for further studies, primary human CPEC dissociation cultures showed growth capacity even after cryopreservation at −80 °C.

### Characterization of CP cell types

With the dissociation approach, we aimed to establish a homogeneous human CPEC culture. However, other cell types are present in the CP. Based on single cell analysis for the mouse CP, Dani et al. recently provided a cell atlas identifying six different cell types in the CP including epithelial, mesenchymal (fibroblasts), endothelial, immune (macrophages), neuronal and glial cells (Dani et al., 2021). Additionally, evidence from murine studies suggests that proliferative CP cells have stem cell properties giving rise to neurons and glia (Li et al., 2002; Bolos et al., 2013). We therefore investigated the cultures by morphology, applying CPEC-specific markers, and by the expression of functional proteins. The cells showed typical cuboidal morphology which was further highlighted by labeling the cytoskeleton (vimentin and cytokeratin). TTR is a thyroxin transport protein widely used as a specific marker for CPEC (Zheng et al., 1998; Southwell et al., 1993; Delery and MacLean, 2019; Schroten et al., 2012; Tsutsumi et al., 1989) which we found ubiquitously expressed, even more homogeneous in culture than in the dissected *post mortem* tissue. Macrophages known to occur in the CP (Kierdorf et al., 2019) were the most abundant cell type in the stroma in our human CP samples. The suggested application of AraC (Strazielle and Ghersi-Egea, 2011; Tsutsumi et al., 1989; Schroten et al., 2012) had the clear effect to prevent growth of macrophages. Moreover, the control cultures without AraC appeared overgrown after several weeks *in vitro*. Since stains for GFAP, CD31, and HuCD were negative, we can exclude astrocytes, endothelial cells, and neurons, respectively in the cultures. Taken together, these observations indicate that the proliferating cells in the newly established human dissociation cultures are most likely differentiated CPEC. In addition, analyzing the expression of functional proteins, we found ZO-1 expressed at cell–cell contact sites, indicating the formation of tight junctions between the cells, characteristic of CPEC (Wolburg et al., 2001). Dissociated CPEC were also positive for AQP1, NKA and NKCC1 although the expression patterns were different from *in vivo* stains.

### Effects of substrate coating and medium on dissociation cultures

CPES are connected to a basal lamina *in vivo.* To optimize cell growth, we tested the cells’ behavior on collagen and laminin substrate. Cells on collagen coating showed better initial adherence and a more heterogenous morphology. In contrast, cells on laminin-coated surfaces exhibited a predominantly cuboidal morphology and slower attachment. These findings are similar to observations of previous CP culture studies, for example, Strazielle et al. hypothesized that laminin coating may favor the selection of epithelial cells in CP cultures from newborn rats (Strazielle and Ghersi-Egea, 1999). Our data suggest that the substrate influences cell morphology and growth patterns but we cannot exclude that cell types other than CPEC were present at the early stages in collagen coated dishes. Consistent with our observation, Delery et al. reported a selective attachment of CPEC on collagen with poor adherence for contaminating cells such as fibroblasts (Delery and MacLean, 2019). Nevertheless, immunostains and the morphology in later stages showed no substantial differences between cells cultured on collagen or laminin. We suggest that both coatings are equally suitable for the investigation of functional mechanisms although the choice of coating may be relevant in other research fields.

Interestingly, a study on primary porcine CP cultures suggested that the cultivation in serum-free medium enhanced cell contacts and polarity (Hakvoort et al., 1998). Exposing the human primary cultures to serum-free medium, we observed a decrease in cell growth that we could not compensate with the addition of growth factors. Since there was no substantial difference in the expression of characteristic CPEC-proteins we think that serum withdrawal may not be necessary for the complete differentiation of primary human CPEC *in vitro.* Strazielle et al. pointed out that the effect of serum-free medium has only been reported in porcine CP, whereas primary cultures from other species show differentiation in serum-supplemented medium (Strazielle and Ghersi-Egea, 2011).

### Aquaporin expression in CP cultures

As we previously discovered AQP4 in human *post mortem* CPEC (Deffner et al., 2022) we were interested whether the cultured CPEC would express this water channel as well. In line with previous observations, we found that several of the AQP4-positive CPEC displayed a basolateral, polar AQP4 expression pattern in the explants. In the dissociation culture, however, AQP4 expression was restricted to cell borders, in contrast to the cytoplasmatic expression pattern of AQP1, suggesting a potential interaction of AQP4 with specific membrane-associated proteins that do not interact with AQP1. Although the expression of AQP4 positive cells varied in the dissociation cultures, we found such cells in cultures from all three donors (Supplementary Figure 3). Previous studies have shown that specific components associated with the dystrophin–dystroglycan complex, such as agrin and laminin, induce AQP4 clustering in astrocyte cell cultures (Noël et al., 2020a; Noell et al., 2007; Noël et al., 2020b), and the loss of dystrophin disrupt AQP4 polarity in murine astrocytes (Belmaati Cherkaoui et al., 2021). In this study, we tested whether laminin and collagen substrate have different effects on AQP4 expression patterns in CPEC. However, we found the membranous expression pattern of AQP4 at cell–cell contact sites on both laminin and collagen coatings. This is nevertheless consistent with our previously reported staining results (Bihlmaier et al., 2023) of fixed CP tissue that showed no correlation between the occurrence of AQP4 positive cells and laminin nor with *β*-dystroglycan. In addition, dystrophin immunoreactivity was absent in the CP but present on astrocytic endfeet of adjacent subependymal tissue (Bihlmaier et al., 2023). This finding and our culture data suggest an underlying mechanism of AQP4 regulation in CPEC different from the polarized expression in astrocytes. Possibly for CPEC, intracellular regulators such as protein kinase-A and calmodulin play a more important role in AQP4 cell surface localization as suggested by *in vitro* studies (Kitchen et al., 2020) in astrocytic endfeet.

### Limitations

One difficulty of this culture model is the limited availability of human body donors, who also vary in *post mortem* time and age. In addition, interpretation of the results obtained from primary culture models can be challenging as they can exhibit substantial variability, e.g., in protein expression patterns.

## Conclusion

In summary, by establishing a primary culture model from human body donor CP, we provided an *in vitro* model that showed protein expression similar to uncultured human cells, although we observed loss of polarity in culture. Both, fixed and cultured CPEC, showed positive immunofluorescence signals for the proteins TTR, actin, vimentin, ZO-1, AQP1, NKA, NKCC1. In addition, we detected the expression of AQP4 *in vitro* as *in vivo*. We therefore suggest this model to be a valuable tool to investigate functional mechanisms in human CPEC especially in the context of aging and neurodegenerative diseases.

## Data Availability

The raw data supporting the conclusions of this article will be made available by the authors, without undue reservation.

## Supplementary material

SUPPLEMENTARY FIGURE 1 Transmitted light image of a human CP explant culture on a PTFE membrane insert at three different time points *in vitro*. At the border of the explant, groups of cells (magnification of the boxed areas) show a changing, dynamic pattern, apparent at 14 and 30 days *in vitro*.

SUPPLEMENTARY FIGURE 2 **(a)** Virtually all CPEC in dissociation cultures stained positive for Pan-Cytokeratin, indicating epithelial characteristics. Most cells were also positive for Transthyretin (TTR). **(b)** Many cells were also positive for NKCC1 although the expression pattern was different from *in vivo* stains. Cells were derived from body donor H103 in **(a)**, and H102 in **(b)**. Scale bars: 50 μm.

SUPPLEMENTARY FIGURE 3 Dissociated cells *in vitro* from CP tissue of three body donors. In all three cultures, cells were positive for ZO-1, although strands were discontinuous. We found a high variability of AQP4 expression reflecting the heterogeneous expression observed *in vivo* (Deffner et al., 2022) yet we found AQP4 positive cells in cultures from all donors (arrows). AQP4 was often close to the apical ZO-1 expression (arrows, see Figure 8). For quantification see Supplementary Table 1. The panel on the right is a negative staining control omitting primary antibodies.

SUPPLEMENTARY FIGURE 4 Dissociated cells were negative for the astroglial marker GFAP, the neuronal marker HuCD, and endothelial marker CD31.

SUPPLEMENTARY FIGURE 5 Dissociated cells of body donor 101 of the fourth ventricle at 19 resp. 23 days in vitro (div) showed overall slower growth than cells from the lateral ventricles. Scale bar 100 μm.

SUPPLEMENTARY FIGURE 6 Dissociated cells of body donor 103 that had been cryopreserved and re-thawed. **(a)** 5 days, **(b)** 8 days after replating. Scale bars 100 μm in **(a)**, and 50 μm in **(b)**.

SUPPLEMENTARY TABLE 1 Cell counts of ZO-1 and AQP4 stained cells in dissociation culture. Fields of views (450 μm x 350 μm) of ZO-1 positive cells were analyzed for AQP4 immunofluorescent signals (see Figure 8 and Supplementary Figure 3). The percentage of AQP4 positive cells ranged from 3.8% up to 75%. This indicates the heterogeneity of AQP4 expression as is observed *in vivo*.
