# Supplementary material for: Primary cell and tissue cultures of human choroid plexus epithelial cells
Source: Front Cell Neurosci. 2026 Aug 31;20:1827493. doi: 10.3389/fncel.2026.1827493 (PMC13572275; doi:10.3389/fncel.2026.1827493)
Supplement: Supplementary file 1 [file Table_1.DOCX]

|  | ZO-1+cells | AQP4+cells | AQP4/ZO-1 cells (%) |
| --- | --- | --- | --- |
| H101 | 32 | 4 | 12.5 |
|  | 53 | 2 | 3.8 |
|  | 29 | 5 | 17.2 |
|  | 47 | 2 | 8.5 |
| H102 | 51 | 22 | 43.1 |
|  | 32 | 16 | 50 |
|  | 24 | 15 | 62.5 |
|  | 52 | 21 | 40.4 |
|  | 26 | 19 | 73,1 |
|  | 33 | 7 | 21.2 |
|  | 35 | 13 | 37.1 |
|  | 23 | 27 | 75 |
| H103 | 92 | 9 | 9.8 |
|  | 40 | 7 | 17.5 |
|  | 58 | 5 | 8.6 |
|  | 74 | 10 | 13.5 |
|  | 98 | 11 | 11.2 |
|  | 122 | 15 | 12.3 |
|  | 45 | 25 | 55.6 |
|  | 86 | 3 | 5.7 |
